# The sterile society: hyperselection and the loss of adaptive variance

**DOI:** 10.3389/fpsyg.2026.1904076

**Published:** 2026-08-19

**Authors:** Jose C. Yong, Simon S. Groome, Edison Tan

**Affiliations:** 1 School of Social and Health Sciences, James Cook University Singapore, Singapore, Singapore; 2 School of Social Sciences, Singapore Management University, Singapore, Singapore

**Keywords:** efficiency, evolutionary mismatch, homogeneity, hyperselection, proxy optimization, proxyeconomics, self-organization, variance

## Abstract

Self-organizing systems utilize information to maintain ordered states. With competitive pressure, these systems optimize toward proxies of their goals—tractable indicators that are informationally useful because they simplify complex objectives, resulting in the survival and prevalence of systems that are most efficient at pursuing proxy targets. While proxy optimization enhances short-term success, it progressively strips away variance upon which long-term adaptation, resilience, and human flourishing depend. Modern technologies escalate these dynamics to intensities without clear evolutionary precedent, operating as a form of hyperselection on proxy-maximizing strategies which lead to increasingly formulaic, homogenized, and sterile environments. The detrimental impact of hyperselection and an overly optimized world—from pop culture and academia to urban design and wellbeing—are discussed in terms of their active role in creating evolutionary mismatch and the importance of seemingly “inefficient” processes as sources of adaptive input.

## Introduction

A kind of discontentment has recently sprouted across domains of human activity. Television and film commentators have argued that “television shows are getting stupider” as entertainment platforms like Netflix increasingly default toward safe, predictable productions optimized to capture audience engagement rather than producing distinctive or enduring shows (Chilton, 2026). In music, scholars and critics alike have observed that songs sound increasingly formulaic, similar, and unlikely to achieve lasting cultural “legacy” status (Kullick and Petry, 2026; Lech et al., 2025; Thompson, 2014). In professional football, fans and pundits lament that games appear increasingly tactically rigid and mechanical, with players operating within systems that discourage individual flair or improvisation (Allnutt, 2025; Hugh, 2024). And in academia, research is undergoing a process of “junkification” as scientific ecosystems increasingly reward outputs that optimize success metrics (e.g., publication counts, citations, journal rankings, grant volume) rather than epistemic progress, resulting in an inundation of papers that add little value (Groome et al., 2026; Rhodes and Linnenluecke, 2025). This diagnosis echoes earlier critiques, such as Bruce Charlton’s (2009) question of why modern scientists appear increasingly risk-averse, unoriginal, and dull.

Although these concerns emerge across seemingly unrelated domains, they reflect an underlying dynamic whereby systems operating under intense competitive pressure increasingly converge upon strategies that efficiently maximize measurable outcomes (Braganza, 2022). This process of “hyperselection” favors highly optimized but homogeneous behavior and production, creating a world that is increasingly predictable, mediocre, and sterile. We further suggest that these same dynamics underlie more ominous issues related to physical and mental health, as hyperselection erodes the variance, diversity, and richness that human individuals and collectives as self-organizing systems feed on for long-term adaptation and thriving (Lukianoff and Haidt, 2018). While evolutionary approaches to psychology and behavior have traditionally focused on understanding the evolved mechanisms underlying human functioning and wellbeing, we suggest that perspectives from information science and proxyeconomics can provide additional insight into how modern optimization pressures affect the conditions those mechanisms evolved to rely on, thereby shedding new light on the dynamics underlying human resilience and flourishing.

## Hyperselection

Information theory is an important yet underutilized complement to evolutionary psychological and behavioral science because adaptive challenges are fundamentally informational problems (Adami, 2024; Yong, 2025). Organisms must identify fitness-relevant opportunities and threats, distinguish signal from noise, and allocate finite resources under conditions of uncertainty. From this perspective, biological systems are information-processing entities that resist entropy by extracting structure from their environments. This capacity for self-organization depends on the efficient coordination of energetic resources to maintain bodily integrity, preserve ordered states, and replicate (England, 2013). Because self-organizing systems operate under selective pressure, they tend toward strategies that allow adaptive goals to be achieved without unnecessary expenditure of energy or resources (Corning, 2025).

Recent theoretical work on proxyeconomics has highlighted how agents within competitive systems pursue goals by optimizing on proxies for those goals rather than the goals themselves (Braganza, 2022; Braganza et al., 2024). Such proxy use emerges naturally from the constraints faced by self-organizing systems. Directly optimizing complex, multidimensional goals is often computationally and energetically prohibitive, so systems gravitate toward simpler indicators that compress complexity into informationally efficient, tractable signals that correlate with desired outcomes (Braganza et al., 2024). Such proxies enable living systems—from individual organisms to human institutions—to coordinate action more efficiently by simplifying decision-making and reducing the costs associated with pursuing complex goals. This work has primarily focused on how optimization on proxies progressively decouples them from the goals they were intended to represent as behaviors that maximize the performance metric rather than the underlying objective are favored, resulting in metric corruption (Strathern, 1997; John et al., 2024)—a phenomenon encapsulated by Goodhart’s law: “When a measure becomes a target, it ceases to be a good measure”.

Another important consequence that has received less attention in this work is the effect of proxy optimization on convergence, homogenization, and the loss of *adaptive* var*iance*. Competitive pressures reward strategies that most efficiently maximize proxies, thereby culling “inefficient” strategies and restricting the diversity of viable pathways for pursuing goals. In the short run, this can generate impressive gains as systems become more lean, ordered, and effective at achieving proxy-correlated outcomes. Yet many complex systems also depend on richness and diversity as inputs that sustain resilience, innovation, and enduring functionality. Natural selection, for instance, favors functional polymorphism, which ensures that species persist in the face of varied challenges to survival and reproduction within an ecosystem (Leimar, 2005). Variance, in this sense, is adaptive material. When selection pressure becomes extreme, however, agents that deviate from proxy-optimizing strategies incur severe costs while only those who conform can persist, causing the system to gradually converge on a small set of behaviors that satisfy the proxy while neglecting other forms of value that are not captured by it.

This convergence-producing tendency has been highlighted across a variety of theoretical traditions. In evolutionary biology, for instance, stabilizing selection (Schmalhausen, 1949) and Fisherian runaway models (Crespi et al., 2022; Fisher, 1930) describe how selection can reduce variation or produce extreme traits over biological timescales. Related dynamics have been formalized in evolutionary game theory, where replicator models show how strategies that yield higher relative payoffs increase in frequency, displace alternatives, and converge on a narrow range of evolutionarily stable outcomes (Maynard Smith, 1972; Maynard Smith and Price, 1973). In the organizational domain, institutional isomorphism captures how coercive, normative, and mimetic pressures drive organizations to adopt similar structures and practices (DiMaggio and Powell, 1983; Frumkin and Galaskiewicz, 2004). Cultural evolutionary perspectives likewise explain how conformist transmission, prestige-biased learning, and cultural attractors cause populations to converge on shared behaviors and traditions over time (Boyd and Richerson, 1985; Claidière et al., 2014; Henrich, 2001). Studies on exploration–exploitation trade-offs in reinforcement learning, organizational behavior, and evolutionary ecology also reveal how over-commitment to exploitation can lead systems to settle prematurely on a few familiar solutions and reduce the diversity of options available for future challenges (Berger-Tal et al., 2014; March, 1991). Collectively, these various strands emphasize how selection and adaptation come with an intrinsic tendency toward convergence, albeit with comparably less attention paid to the consequences of diminished variance.

We suggest that humans are unique in how far these convergence dynamics can be pushed and, correspondingly, how thoroughly the variance needed to thrive and flourish can be wiped out. Through human ingenuity and technology, selection pressures on proxies can be amplified to evolutionarily unprecedented levels, constituting what we call *hyperselection*: the intense selection of proxy-optimizing strategies that progressively drive systems toward a narrow range of behaviors, designs, or solutions while eroding the adaptive variance upon which those systems ultimately depend. Two mutually reinforcing processes are at play. First, human cognitive and technological capabilities dramatically enhance our ability to identify tractable proxies and develop increasingly precise methods for optimizing them (Yong et al., 2026). Second, these same capacities continually escalate competitive standards, such that what constitutes success becomes an ever-moving target as competitors relentlessly optimize the proxy. Together, these processes progressively remove adaptive variance from human systems—the diversity, alternative pathways, and slower feedback loops that appear inefficient from an optimization standpoint but are essential for exploration, mastery, creativity, and other complex human desiderata to emerge. In doing so, hyperselection strips away the space of viable possibilities through which self-organizing systems develop and express their full potential (Gershenson, 2025; Nesse et al., 2025).

The intensification of selection pressure by modern human interventions can be illustrated through a comparison with earlier eras. Throughout most of human history, optimization was constrained by limited information, high transaction costs, and various forms of lag. A medieval merchant, for instance, could pursue profit, but the speed and precision with which he could identify and exploit opportunities were bounded by the rudimentary technologies available to him. Competing merchants faced similar limits, so the standards for success could not be ratcheted up indefinitely. Mate selection likewise historically unfolded within offline settings that constrained both the number of potential partners that could be accessed and the efficiency with which they could be evaluated (Goetz et al., 2019). Schools, workplaces, religious communities, and shared activities provided relatively slow but multifaceted contexts in which people could observe one another across situations, assess compatibility, and allow attraction and trust to develop gradually through repeated interaction. Modern innovations dismantle much of this “slack.” Dating platforms, for example, allow users to communicate with many potential partners and compare them on the basis of a small set of highly observable traits (e.g., physical attractiveness, wealth). At the same time, they remove mate selection from the environments in which mates are naturally encountered, replacing messy but informationally rich social settings with highly compressed forms of assessment based on curated profiles and brief interactions. The result is a system that is more efficient but less conducive to forming enduring relationships, thereby contributing to many of the challenges associated with modern romance (Yong et al., 2026).

**Figure 1:**
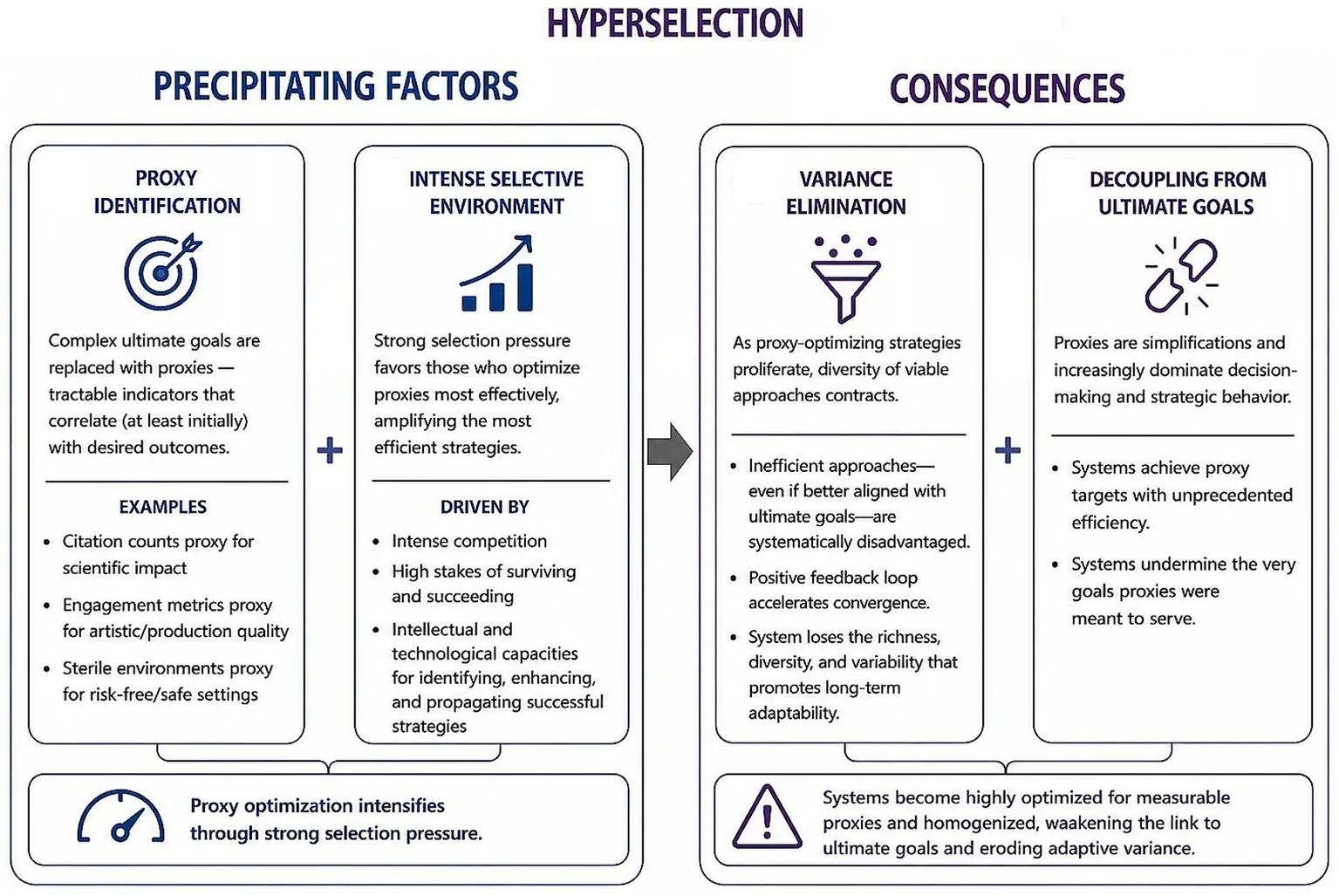
The hyperselection mechanism.

Figure 1 provides a summary of the hyperselection mechanism. To further demonstrate its broad applicability, we highlight a brief selection of examples of how this process and its consequences manifest in human domains.

### Academia

Concerns about declining research quality within the sciences (Chu and Evans, 2021; Smaldino and McElreath, 2016) have prompted discussion of what some scholars have termed “research junkification” (Groome et al., 2026; Rhodes and Linnenluecke, 2025). Indicators of academic competence, such as publication counts, citation metrics, journal prestige, and *h*-indices, increasingly drive institutional incentives. Researchers rationally respond by pursuing projects that maximize measurable outputs, particularly safe but mildly incremental contributions that fit established paradigms and appeal to reviewers, funders, or hirers. The result is large volumes of highly similar work optimized for countable productivity rather than intellectual exploration or transformative insight. High-variance approaches—ambitious projects with uncertain payoffs, heterodox methodologies, or unfashionable questions—become systematically disadvantaged. The academy succeeds by its own metrics while undermining the epistemic diversity on which scientific progress depends (Charlton, 2009; Park et al., 2023).

### Popular culture and media

Analogous pressures operate in cultural production. Critics have argued that contemporary film, music, and streaming content increasingly rely on repetitive formulas designed to maximize engagement and profitability (Hesmondhalgh, 2026; Kullick and Petry, 2026). Armed with advanced production tools, producers identify patterns that reliably attract audiences (e.g., familiar narrative structures, proven melodic sequences, algorithmic recommendations optimized for attention) and ruthlessly exploit them. From a proxy standpoint, the system performs admirably as consumption metrics rise and revenues increase. Yet audiences frequently report a diffuse sense of dissatisfaction that content has become predictable, mediocre, and less meaningful despite its technical sophistication, as the variance that characterizes novelty and creative risk has been streamlined away (Hesmondhalgh, 2022).

### Sports

Similar dynamics have been observed in professional sport, particularly in complex team games where collective organization can supplant individual brilliance. In football, the intensified pursuit of measurable outcomes—goals, wins, league positions, and especially the financial stakes they determine—has encouraged increasingly conservative and tactically optimized approaches (Wilson, 2024). Teams adopt increasingly obsessive approaches to nutrition and recovery, minimize risky play, and prioritize collective solidity over individual flair. Arsenal’s 2025–26 Premier League title exemplifies this tension. The club ended a 22-year drought through ruthless set-piece dominance, scoring more dead-ball goals than any team in Europe’s top five leagues, and attracted widespread criticism for killing the game. Critics have taken to calling such approaches “haramball,” football so cynical it feels sinful to watch (Hugh, 2024).

### Cities and urban spaces

Modern cities have been described as looking increasingly alike despite their geographical and cultural differences (Anderson, 2020). Historically, cities developed distinctive identities through the gradual accumulation of local traditions, architectural styles, businesses, institutions, and patterns of social interaction. By contrast, contemporary urban environments shaped by pressures such as commercial viability, tourism, and foot traffic converge into standardized forms that can be easily replicated and scaled (Nguyen, 2022; Relph, 1976). Local businesses and neighborhoods are replaced by the same Starbucks, Apple stores, luxury-brand outlets, hipster coffee shops, and lifestyle developments that have become ubiquitous from Seoul to Milan to New York. Although such environments are often easier to navigate and commercially successful, they contribute to an “unbearable sameness of cities” whereby urban spaces become increasingly interchangeable, less memorable, and detached from the local histories and communities that once gave them their unique character (Schwindt, 2018).

### Social status

Social status is particularly prone to proxy optimization given how important yet nebulous it is. Status can be attained in various ways including competence, generosity, wisdom, leadership, craftsmanship, and service to one’s community, and society benefits from diverse pathways to status because a wider range of abilities can be harnessed while allowing individuals with different talents to achieve social value and esteem (Yong et al., 2024). Competitive modern environments, however, produce fixations on highly visible and measurable indicators such as wealth, occupational prestige, educational credentials, attractiveness, and online popularity. As competitive pressures intensify around these indicators, alternative status pathways become devalued, resulting in decreased status diversity and the neglect of qualities that are hard to quantify yet crucial for collective functioning. Hyperselection on status proxies also fosters status anxiety, materialism, and other obsessive status-seeking behaviors, which come at the expense of relationships, community engagement, and other activities that underlie sustained wellbeing (Yong et al., 2026).

### Health and wellbeing

Perhaps the most pressing implications of hyperselection emerge in the domain of health and wellbeing. The hygiene hypothesis proposes that exposure to diverse microbial environments contributes to the training and proper calibration of the immune system (Perkin and Strachan, 2022; Rook, 2013; Strachan, 1989). Immune systems evolved against an ancestral backdrop of microbial richness and variability, conditions that were not merely tolerated but integral to healthy immune development. However, the pursuit of safety as a survival instinct has driven contemporary societies toward increasingly sanitized and sterile environments. Medical technologies (e.g., antibiotics, disinfectants) and modern hygienic practices effectively decrease pathogenic threats, but in doing so, also reduce the microbial diversity that immune systems evolved to expect. The resulting paradox is that systems optimized for protection and cleanliness may inadvertently contribute to immune dysregulation, including autoimmune conditions, allergies, and inflammatory disorders (Bach, 2002; Haahtela et al., 2013).

A similar logic applies to psychological functioning and wellbeing. Human cognitive and emotional systems evolved within environments characterized by substantial variability in social interactions, physical demands, uncertainty, and risk, which serve as formative inputs shaping resilience and other adaptive capacities (Buss, 2000). Modern environments, however, increasingly optimize for comfort, convenience, and psychological safety as proxies of survival and fitness. While it is often difficult to view such optimizations as undesirable because their immediate benefits are obvious (Yong and Kanazawa, 2026), they reduce exposure to the kinds of variability and challenges that human psychological systems evolved to learn from, leading to more deep-seated issues (Lukianoff and Haidt, 2018). Reduced exposure to hardship may weaken resilience; curated social environments may diminish tolerance for interpersonal conflict and ambiguity; algorithmically hyper-optimized entertainment and stimulation may displace less immediately rewarding yet richer experiences (e.g., going into nature); and increasingly sophisticated information technologies (e.g., artificial intelligence) may decrease the need to exercise memory, process information, or think critically. As such, hyperselection may create highly efficient and safe environments that ironically undermine optimal human development by eliminating the richness and diversity of inputs needed to foster and maintain adaptive psychological functioning.

### Artificial intelligence technologies as evolutionarily novel hyperselection engines

The hyperselection framework also affords an opportunity to contextualize the rapid emergence of modern artificial intelligence (AI) tools, including recommendation algorithms, search engines, large language models (LLMs), and other generative technologies as the most evolutionarily novel or advanced hyperselection engines developed to date. Unlike earlier technologies, AI technologies do not require human intervention to identify and optimize successful strategies; they continuously update their own criteria for success based on user interaction patterns and behavioral data. The outputs of one optimization cycle (e.g., highly engaging posts, frequently clicked links, successful prompts, widely shared AI-generated content) become inputs to the next, further refining what is surfaced and replicated (Amodei et al., 2016; Chaney et al., 2018; Jiang et al., 2019).

Over time, this recursive optimization narrows exploratory search spaces and channels attention toward an increasingly restricted set of proxy-maximizing patterns, thereby further simplifying informational ecologies and reducing exposure to alternative ideas, strategies, and forms of expression (Chaney et al., 2018; Glickman and Sharot, 2025; Sourati et al., 2026). People may come to find that everything from online content and entertainment to news, advice, and creative work begins to look, sound, and feel increasingly alike, despite having access to an unprecedented volume of information. As such, AI functions not merely as another participant in hyperselection but as an infrastructure that recursively accelerates hyperselection itself, with profound downstream consequences for human cultural and informational environments.

## Discussion

As adaptive systems must achieve complex goals under conditions of uncertainty and limited information, informationally useful proxies that compress complexity into tractable signals are favored. From an information-theoretic perspective, both biological and cultural evolution are processes that use information to identify and retain patterns associated with adaptive outcomes—natural selection favors genes that correlate with reproductive success, while cultural evolution selects for behaviors, ideas, and institutions that correlate with culturally valued outcomes and, ultimately, reproduction at a deeper level (Adami, 2024; Henrich and McElreath, 2007; Yong, 2025) Optimization is therefore an inevitable feature of self-organizing systems utilizing information to pursue adaptive goals that underlie ordered states more effectively.

Competitive pressures have always existed, but they operated alongside tradition, chance, local contingencies, slower feedback cycles, and other forms of friction. Contemporary technologies remove these inefficiencies, enabling selection on proxies to reach unprecedented levels. As systems become increasingly organized around highly proxy-optimized strategies, variance in behaviors and strategies is reduced. Yet what appears wasteful or suboptimal through the lens of optimization may be a necessary component of adaptive functioning for many systems. Hyperselection therefore creates a homogenization paradox as systems become increasingly efficient according to their own metrics while simultaneously removing the variance upon which long-term adaptation depends.

Resolving this paradox requires recognizing that not all valuable outcomes can be captured by tractable indicators, despite the benefits of optimization and efficiency. Some goods, such as trust, creativity, meaning, insight, and discovery, are difficult to simplify without losing vital information. Moreover, if technological amplification allows optimization to operate at evolutionarily novel intensities, then human systems may be deprived of adaptive variance at rates that exceed their capacity for self-correction. Variance-preserving mechanisms may therefore become an increasingly important design principle for social, cultural, and institutional systems if we wish to protect the features associated with thriving human ecologies. For instance, academic institutions may benefit from promoting exploratory research alongside metrics-driven science; educational systems from maintaining diverse pedagogical approaches rather than standardized testing; and cities from preserving local heritage over maximizing commercial gains. A greater emphasis on mastery over performance may go a long way toward counterbalancing increasingly hyperselective environments.

### Juxtaposing hyperselection against similar frameworks

The present framework provides an information-theoretic integration of existing perspectives describing how selection pressures promote convergence, together with recent work on proxyeconomics demonstrating how optimization around proxies can decouple them from the outcomes they were originally intended to represent (Braganza, 2022; John et al., 2024). Extant frameworks have largely been developed within specific domains (e.g., genes, cultural traditions, organizational forms, behavioral strategies) emphasizing different units of selection and mechanisms through which convergence occurs (e.g., Claidière et al., 2014; DiMaggio and Powell, 1983; Maynard Smith, 1972; Scott, 1999). By contrast, hyperselection identifies a general process whereby proxy optimization and competitive selection progressively erode adaptive variance across biological, cultural, technological, and institutional systems.

The framework further emphasizes a form of selection that is evolutionarily novel in intensity. Whereas existing perspectives generally describe convergence under ordinary selection pressures, hyperselection focuses on contexts in which satisficing gives way to relentless proxy-maximization, an intensity made possible by human intellectual and technological capacities. Modern humans are therefore uniquely susceptible to hyperselection because optimization can now proceed at evolutionarily unprecedented extents that outpace more natural tempos of change (e.g., biological selection). Finally, hyperselection foregrounds homogenization and the erosion of adaptive variance as distinct system-level consequences of intense optimization, highlighting the importance of preserving the inefficiencies needed to sustain high-quality human systems.

### Implications for evolutionary approaches to psychology and behavior

The hyperselection framework advances evolutionary psychological and behavioral theory in several ways. First, it extends evolutionary mismatch theory—the view that many modern pathologies arise because evolved physiological and psychological mechanisms encounter environments that differ from those in which they were selected and calibrated (Li et al., 2018)—beyond a static distinction between ancestral and modern environments. Specifically, it emphasizes how technological amplification not only changes the rate and structure of selection processes but also reshapes the informational inputs upon which evolved mechanisms depend (Yong et al., 2026).

Second, the framework identifies adaptive variance as one such informational input and, more broadly, as a vital environmental resource for evolved systems rather than a mere by-product of imperfect optimization. Organisms—including humans—likely evolved to not only tolerate an imperfect world but also benefit from it. Just as physiological development depends on appropriate nutritional and microbial diversity, psychological development similarly requires exposure to diverse challenges, social contexts, and experiential inputs. Likewise, exposure to diverse ideas, cultures, architectural styles, scientific perspectives, and artistic formats may enrich the cognitive and cultural environments from which inspiration and innovation arise. The uncertainties and rough edges of everyday life therefore provide many of the varied informational inputs through which resilience, coping, and other forms of adaptive functioning are cultivated.

Finally, the hyperselection framework helps bridge ultimate and proximate levels of explanation by showing how optimization around proximate proxies (e.g., comfort, safety, status, engagement) can systematically undermine the ultimate adaptive functions those proxies were originally correlated with (e.g., resilience, survival, reproduction). More broadly, by integrating perspectives from evolutionary biology, information theory, proxyeconomics, and related theories of selection and convergence, the present framework provides a consilient lens through which diverse biological, psychological, cultural, technological, and institutional phenomena can be understood as manifestations of a common evolutionary process (Yong, 2025).

### Caveats

It is important to recognize that proxy optimization is not inherently problematic. Proxies exist because they simplify complexity, enable coordination, and provide feedback that guides adaptive decisions and behaviors (e.g., Dmitriev and Wu, 2016; Peters et al., 2013; see also Prentice, 1989). Without tractable indicators, many large-scale systems would struggle to function; for instance, markets cannot allocate resources properly, and agents and organizations cannot make decisions fast. The efficiency gains from proxy optimization are real and often substantial, and some systems are even designed to operate under intense optimization pressures, such as machine learning models which are engineered to converge rapidly on high-performing solutions, industrial processes which benefit from standardization, and safety-critical systems which prioritize reliability over exploration.

Rather than treating proxy use or convergence as undesirable in themselves, the present perspective draws attention to the boundary conditions under which intense optimization becomes self-undermining. As highlighted in our scientific, artistic, urban, and psychosocial examples, hyperselection is especially problematic for systems whose optimal, long-term functioning depends on having room for cultural, behavioral, or informational diversity to be maintained. These systems derive their robustness, creativity, and adaptive capacity from a broad repertoire of strategies, norms, ideas, and experiences. When this diversity is eroded, further optimization yields diminishing and eventually negative returns as the adaptive variance upon which the system depends is stripped away (see Scott, 1999).

## Conclusion

There is something enchanting about places, activities, and cultures that retain a certain rawness and vibrancy because they have been allowed to develop their own character. A world organized entirely around efficiency and measurable outcomes may be highly ordered and productive, but losing the variation, unpredictability, and imperfections that enrich experience renders such a world increasingly sterile. While proxy optimization is appropriate for industrial or mechanistic systems designed for optimization, many other biological, psychological, and cultural systems depend on preserving diversity and variation as deep sources of adaptation and thus suffer from hyperselection. The challenge is not to abandon measurement or efficiency entirely, but to cultivate a more sophisticated understanding of the costs of optimization and to protect the forms of richness that simplified proxies cannot capture.

## Data Availability

The original contributions presented in the study are included in the article/supplementary material, further inquiries can be directed to the corresponding author.
